# Can Hemp Help? Low-THC Cannabis and Non-THC Cannabinoids for the Treatment of Cancer

**DOI:** 10.3390/cancers12041033

**Published:** 2020-04-23

**Authors:** Farjana Afrin, Mengna Chi, Andrew L. Eamens, Ryan J. Duchatel, Alicia M. Douglas, Jennifer Schneider, Craig Gedye, Ameha S. Woldu, Matthew D. Dun

**Affiliations:** 1 Cancer Signalling Research Group, Medical Biochemistry, School of Biomedical Sciences and Pharmacy, Faculty of Health and Medicine, University of Newcastle, Callaghan, NSW 2308, Australia; Farjana.Afrin@uon.edu.au (F.A.); Mengna.Chi@newcastle.edu.au (M.C.); Ryan.Duchatel@newcastle.edu.au (R.J.D.); Alicia.Douglas@newcastle.edu.au (A.M.D.); Craig.Gedye@newcastle.edu.au (C.G.); 2 Priority Research Centre for Cancer Research, Innovation & Translation, Faculty of Health and Medicine, Hunter Medical Research Institute, New Lambton Heights, NSW 2305, Australia; Jennifer.Schneider@newcastle.edu.au; 3 Centre for Plant Science, School of Environmental and Life Sciences, University of Newcastle, Callaghan, NSW 2308, Australia; Andy.Eamens@newcastle.edu.au; 4 Priority Research Centre for Chemical Biology and Clinical Pharmacology, School of Biomedical Sciences and Pharmacy, Faculty of Health and Medicine, University of Newcastle, Callaghan, NSW 2308, Australia; 5 Calvary Mater Newcastle, Waratah, NSW 2298, Australia

**Keywords:** cannabinoids, cannabidiol, ∆^9^-tetrahydrocannabinol, cannabinoid receptors, G-protein-coupled receptors, cancer, apoptosis, inflammation

## Abstract

Cannabis has been used to relieve the symptoms of disease for thousands of years. However, social and political biases have limited effective interrogation of the potential benefits of cannabis and polarised public opinion. Further, the medicinal and clinical utility of cannabis is limited by the psychotropic side effects of ∆^9^-tetrahydrocannabinol (∆^9^-THC). Evidence is emerging for the therapeutic benefits of cannabis in the treatment of neurological and neurodegenerative diseases, with potential efficacy as an analgesic and antiemetic for the management of cancer-related pain and treatment-related nausea and vomiting, respectively. An increasing number of preclinical studies have established that ∆^9^-THC can inhibit the growth and proliferation of cancerous cells through the modulation of cannabinoid receptors (CB1R and CB2R), but clinical confirmation remains lacking. In parallel, the anti-cancer properties of non-THC cannabinoids, such as cannabidiol (CBD), are linked to the modulation of non-CB1R/CB2R G-protein-coupled receptors, neurotransmitter receptors, and ligand-regulated transcription factors, which together modulate oncogenic signalling and redox homeostasis. Additional evidence has also demonstrated the anti-inflammatory properties of cannabinoids, and this may prove relevant in the context of peritumoural oedema and the tumour immune microenvironment. This review aims to document the emerging mechanisms of anti-cancer actions of non-THC cannabinoids.

## 1. Introduction

The flowering herb *Cannabis sativa* L. (Cannabaceae) has been used in traditional Eastern medicines as an analgesic, anxiolytic, anticonvulsant, sedative, and hypnotic, for almost 5000 years [1]. More recently, medicinal cannabis has been studied for its efficacy in the treatment of epilepsy, inflammation, anxiety, nausea and cancer-related pain [1]. Emerging anecdotal and preclinical evidence has further demonstrated that cannabis can also modulate tumour growth [2]: a finding that has renewed interest in the use of cannabis as a potential anti-cancer therapeutic [3], in spite of the limited available clinical trial data. Encouragingly, the recent rapid change to the legislative status of recreational and medicinal cannabis is now enabling appropriate assessment of the use of cannabis and cannabinoids in the clinical setting [4].

The two main cannabinoids in cannabis are ∆^9^-tetrahydrocannabinol (∆^9^-THC) and cannabidiol (CBD). Each of these cannabinoids demonstrate very different pharmacological actions. Concerns about the undesirable effects caused by the primary psychotropic constituent, ∆^9^-THC, have led to hesitancy to prescribe cannabis for medicinal use. Tachycardia, anxiety, altered cognitive perception, as well as other behavioural issues, are commonly reported symptoms stemming from the use of ∆^9^-THC [5,6]. Further, ∆^9^-THC can also alter immune system function, and increase the susceptibility of a patient to microbial infections [7,8]. In spite of the demonstrated anti-cancer properties of ∆^9^-THC, this cannabinoid has also been revealed to promote tumour growth, invasion and metastasis in some cancer cell types. In breast cancer for example, ∆^9^-THC-mediated increased tumour growth and metastasis was associated with inhibition of the anti-tumour specific immune responses in vivo [8]. A retrospective analysis further demonstrated that cannabis use is associated with the markedly reduced effectiveness of immune checkpoint inhibitors [9]. Therefore, rigorous and meticulous anti-cancer research, both pre-clinically and clinically, is required to provide the necessary medical rationale to support the use of medicinal cannabis as a complementary, or alternative, anti-cancer treatment option.

Conversely, the undesirable side effects, such as drug dependence/tolerance/abuse issues shrouding the use of ∆^9^-THC is not seen with CBD (the second most abundant cannabinoid in cannabis). Indeed, CBD is well-tolerated in patients, even at relatively high doses in contrast with ∆^9^-THC, which has a maximum tolerated daily dose of only 15 to 90 mg in adult patients, and this daily maximum requires ongoing, individualised adjustment, to ensure that side effects are managed for each patient [10]. Furthermore, CBD has anti-psychotic, anti-convulsive, anxiolytic, sedative and anti-inflammatory properties, none of which have been reported for ∆^9^-THC [11]. The more recently reported positive health benefits of CBD, and for other non-THC cannabinoids, has promoted and accelerated research into non-THC cannabinoids [11,12,13]. In spite of these promising findings, legislation continues to lag. For example, the concentration of ∆^9^-THC required by law to classify cannabis as being a ‘low-THC’ variety, varies from country to country. Presently, in the United States, cannabis is permitted for therapeutic use in 24 states and territories, including the District of Columbia, Guam, Puerto Rico and the U.S. Virgin Islands, with an additional 16 states allowing the use of low ∆^9^-THC/high CBD products (commonly referred to as ‘hemp’) for medical reasons, albeit in limited situations [14]. In most Australian states and territories, the limit of ∆^9^-THC in hemp must be below 1% of total plant material [15,16]. This is in direct contrast with the legislation of the U.S. For example, the state law of New Jersey stipulates that a substance with a ∆^9^-THC content of less than 10% (by weight) classifies the substance as low-THC [17]. This highlights the significant variation in global cannabis classification systems, and as such, creates considerable complexity when attempting to reveal the clinical efficacy of low-THC cannabis and non-THC cannabinoids.

### Effects of Cannabis on Human Health

Medicinal cannabis is increasingly being used in the clinical setting [18,19,20,21]. However, contrasting views on the long- and short-term impacts on patient health, following the sustained use of ∆^9^-THC cannabis, persist. There is no doubt that recreational use is associated with substantial adverse health effects. Still, the burden of disease stemming from the recreational use of cannabis is much lower than other commonly used legal and illicit substances [22]. Reports of brain damage and cognitive dysfunction [23] are contrasted by clinical studies that show improved cognitive function with the controlled use of ∆^9^-THC cannabis [24]. Furthermore, ∆^9^-THC and synthetic cannabinoids (sCB) (discussed in Section 2), protected normal rat astrocytes from induced cell death post exposure to ceramide (N-acetylsphingosine cell-permeable analogue; see Section 3.4.1), via the activation of phosphatidylinositol 3-kinases –PI3K/protein kinase B–Akt/p90 ribosomal S6 kinase–RSK, survival signalling pathways [25]. Conversely, Lenzi et al. showed that sCBs have mutagenic capabilities in healthy human lymphoblasts and can cause chromosomal damage at the concentration range of 25 to 50 µM [26]. Koller et al. also demonstrated that sCBs can cause DNA damage in human lymphocytes [27].

There is unanimous support to encourage harm minimisation approaches in order to decrease medicinal or recreational cannabis use while pregnant or postpartum [28]. For example, ∆^9^-THC containing cannabis use is negatively correlated with foetal birth weight (up to a 450 g reduction) [29], and the development of neurotransmission systems, with the endocannabinoid system playing a number of important roles in brain development. Although the evidences so far are ambiguous, it is unsurprising that prenatal exposure to cannabis may have neurocognitive consequences, including affective mental disorders (anxiety and depression), and attention deficit hyperactivity disorders (reviewed in [30]), which are also common occurrences in the adult life of long-term ∆^9^-THC cannabis users (reviewed in [31]). Interestingly, CBD elicits different neurocognitive activity compared with that of ∆^9^-THC [32]. In some cases however, CBD can induce somnolence and fatigue [33], akin to the use of ∆^9^-THC. CBD also has the ability to cross the placenta and to reach breast-milk, further highlighting the need to abstain while pregnant or when postpartum [33]. Due to the risk of accumulation, CBD dose adjustment is required in patients with hepatic impairment [34]. CBD may cause hepatotoxicity, as mouse modelling showed that an acute dose of 2460 mg/kg (24 hr), or a sub-acute dose of 615 mg/kg (10 days), increased markers of liver damage, including liver-to-body weight ratios, plasma alanine aminotransferase and aspartate aminotransferase enzyme levels, and total bilirubin after 3–4 days of treatment [35]. Furthermore, there is currently limited evidence regarding the long-term safety and cognitive consequences of the sustained consumption of CBD, either in the recreational or medicinal setting [36].

## 2. Cannabinoids

To date, over 620 chemical compounds, including cannabinoids, cannabis-specific prenylated flavones (cannflavin A and B), and terpenoids, have been isolated from traditional cannabis [37]. Unlike recreational varieties of the cannabis plant that contain high levels of ∆^9^-tetrahydrocannabinolic acid (the biosynthetic precursor of ∆^9^-THC), the primary cannabinoids in low-THC cannabis/hemp include cannabidiolic acid (CBDA), cannabigerolic acid (CBGA), cannabichromenic acid (CBCA), and their decarboxylated forms cannabidiol (CBD), cannabigerol (CBG), and cannabichromene (CBC), respectively [37,38].

Cannabinoids (a group of terpenophenolics) are highly abundant in this plant species, with more than 100 different cannabinoids identified as constituents of the cannabis plant [37]. Endogenous and exogenous cannabinoids can be separated into three main groups, including; (1) endocannabinoids (eCBs); (2) phytocannabinoids (pCBs), and; (3) sCBs [39]. The eCBs are an endogenous group of lipid-based, retrograde neurotransmitters that bind to cannabinoid receptors (CBRs) and related receptor proteins in animals [40]. There are two major human eCBs, anandamide (AEA) and 2-arachidonoylgycerol (2-AG) [41]. Both AEA and 2-AG are derived from the non-oxidative metabolism of membrane phospholipids [42], with AEA degraded by the fatty acid amide hydrolase (FAAH) [40], whereas the monoacylglycerol lipase (MAGL) enzyme is primarily responsible for terminal 2-AG degradation [43]. pCBs (or simply cannabinoids) are naturally occurring plant-derived cannabinoids unique to the cannabis plant, with the two major chemicals derived from the common precursor, cannabigerol (CBG) [44]. sCBs, such as WIN-55, 212-2, HU120, JWH-015 and JWH-133, are classified on the basis of their chemical structure and affinity for CBRs [39].

## 3. Role of Cannabinoids in Cancer

In vitro and in vivo animal models have shown that cannabinoids have selective anti-cancer activity in a wide range of cancer cell lines, including breast [13], prostate [45], cervical [46], brain [47,48], colon [49] and leukaemia/lymphoma [50,51] (Table 1). A variety of cannabinoids, and cannabinoid extracts, have been subjected to several phase I/II clinical trials (summarised in Table 2). The mechanisms of anti-cancer action for both ∆^9^-THC and non-THC cannabinoids remains enigmatic, however, most studies suggest that ∆^9^-THC induces apoptosis and cytotoxicity via CBR-dependent pathways [50,51,52], while non-THC cannabinoids, such as CBD, modulate the activity of other de-orphan and orphan G-protein-coupled receptors (GPCRs) and non-GPCRs. 

### 3.1. Cannabinoid Receptors (CBRs)

Notably, ∆^9^-THC and sCBs elicit central and peripheral effects via stimulation of the cannabinoid GPCRs, CB1R (*CNR1*) and CB2R (*CNR2*) [63,64], whereas CBD shows negligible affinity for CB1R or CB2R [65]. CB1R and CB2R are expressed on both extracellular and intracellular membranes of organelles, such as the nucleolus, mitochondria and Golgi apparatus [66]. CB1R is primarily found in the brain and in the peripheral central nervous system (CNS), whereas CB2R is predominantly expressed in immune cells [64]. Thus, the involvement of CB1R is more likely to drive psychotropic activity, with the weight of evidence showing that CB2R activation modulates immunological responses [67]. However, CB2R modulated neurological function and its expression in the CNS on microglia, astrocytes, and subpopulations of neurons has also been reported (reviewed in [68]). Nevertheless, both CB1R and CB2R are linked to the anti-cancer effects of ∆^9^-THC [69,70].

### 3.2. Non-THC Cannabinoids Are Ligands for Calcium Selective Ion Channels and Orphaned/De-Orphaned G-Protein-Coupled Receptors

Non-THC cannabinoids, such as CBD, show a reduced affinity for the classical CBRs, acting as an antagonist or inverse agonist of CBRs, with an inhibitory constant (Ki) of 1458.5 ± 158.5 nM for CB1R and 372.4 ± 57.5 nM for CB2R [71]. Therefore, it is unlikely that CB1R and CB2R play a significant role in the anti-cancer effects of CBD. Transient receptor potential vanilloid (TRPV) calcium selective ion channels are targets for cannabinoids [72,73] (discussed in Section 3.2.1). The majority of reports highlight a role for non-THC cannabinoids as ligands for de-orphaned GPCRs, such as G-protein-coupled receptor 55 (GPR55) (discussed in Section 3.2.2) and GPR119. However, GPR119 is only activated by eCBs analogues, including oleoylethanolamide and palmitoylethanolamide, rather than pCBs or sCBs. Therefore, GPR119 is not discussed in detail here, other than to outline that its agonism by specific ligands (such as MBX-2982 or GSK1292263), re-sensitises breast cancer cells that have developed resistance to gefitinib [74]. Uniquely, CBD is a ligand for the orphan GPCRs, GPR3, GPR6 and GPR12 (discussed in Section 3.2.3).

#### 3.2.1. Transient Receptor Potential Channels of the Vanilloid Subtype–TRPV1/2

**Figure 1:**
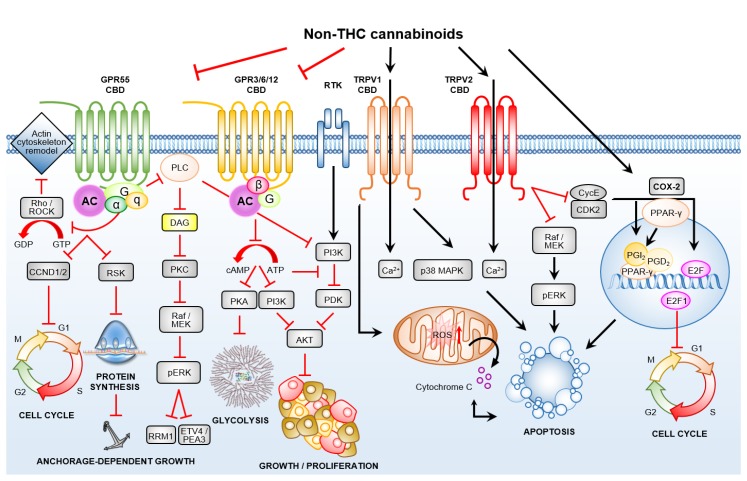
Transient receptor potential channels of the vanilloid subtype proteins and G-protein-coupled receptors mediates the anti-cancer effects of non-tetrahydrocannabinol (non-THC) cannabinoids. Cannabidiol (CBD) antagonises the de-orphaned G-protein-coupled receptor GPR55, and agonise transient receptor potential vanilloids (TRPVs), TRPV1 and TRPV2. CBD may out-compete the natural ligands of orphaned G-protein-coupled receptors (GPCRs), GPR3, GPR6 and GPR12, and this in turn suggests a mechanism for the inhibition of tumour growth and the induction of cell cycle arrest, and/or may even promote selection of cancer stem cells (CSCs).

The transient receptor potential (TRP) superfamily of transmembrane ion channels TRPV1–TRPV4, and TRPA1 and TRPM8, are termed the ionotropic CBRs. The activation of TRPV1 by CBD, and of TRPV2 by CBD and ∆^9^-THC, inhibits human glioma cell proliferation and viability in vitro [58]. TRPV1 receptors regulate calcium influx to trigger apoptosis via the mitochondrial intrinsic and p38 MAPK-dependent pathways (Figure 1) [75]. TRPV1 also degrades EGFR (epidermal growth factor receptor), which is known to be overexpressed and activated in a variety of cancers; findings that highlight the tumour-suppressor capabilities of TRPV1, as well as identifying a role for CBD in cancer cell types where EGFR is overexpressed or mutated [76]. Commensurate with the tumour-suppressor capabilities of TRPV1, the expression of *TRPV1* has been shown to be significantly decreased in the high-grade glioma, glioblastoma multiforme (GBM), thereby potentially reducing the therapeutic benefits of CBD in advanced patients [75]. In addition to TRPV1, TRPV2 plays a role in regulating glioma cell survival and proliferation, with high levels of TRPV2 expression detected in benign astrocytes, with progressively less expression of TRPV2 in high-grade gliomas corresponding with histological grade [77]. Indeed, the repression of *TRPV2* abundance in human glioma cell lines was in turn demonstrated to result in the increased expression of cyclin E1, cyclin-dependent kinase 2 (*CDK2*), transcription factor E2F1 (*E2F1*), RAF proto-oncogene serine/threonine-protein kinase (*RAF1*) genes, and the anti-apoptotic gene, Bcl-xL (*BCL2L1*) [58]. Increased *cyclin E1*, *CDK2*, *E2F1*, *RAF1* and *BCL2L1* gene expression reduced the abundance of the cell death receptors and of proteins encoded by apoptosis-related genes, *Fas* and *procaspase-8*, an expression change that enhanced cell survival and proliferation (Figure 1). Interestingly, CBD has also been shown to enhance the cytotoxicity of certain chemotherapeutics via triggering TRPV2-dependent calcium (Ca^2+^) influx, and this in turn increased the degree of chemotherapeutic uptake, while simultaneously reducing the incidence of chemoresistance in GBM cells. No such effect has been reported for normal human astrocytes (Table 1) [58]. A limitation of this study is, however, that parallel assessment of the TRPV2-dependent Ca^2+^ influx and sensitisation to temozolomide (TMZ), carmustine (BCNU) or doxorubicin (DOXO) was not performed following ∆^9^-THC treatment.

#### 3.2.2. De-Orphaned G-Protein-Coupled Receptor 55

GPR55 is widely expressed in the mammalian brain with its abundance concentrated in large dorsal root ganglions (DRGs) [78], and is also expressed in peripheral tissues such as the endocrine pancreas [79]. GPR55 is a direct target of the pCBs, CBD and ∆^9^-THC, the eCBs, AEA and 2-AG, and the sCB, JWH-015 [63,80]. The downstream effects of GPR55 agonism depend on the cannabinoid present. For example, ∆^9^-THC, AEA, JWH-015, bind GPR55 to drive intracellular release of Ca^2+^ in HEK293 cells, transiently transfected to express human GPR55 (GPR55-HEK293), with this process regulated by G protein, G_q/11_-phospholipase C (PLC) [77]. This effect can be abolished via the transient transfection of HEK293 cells with a dominant-negative mutant of *G_q/11_*, or via use of the CB1R/GPR55 antagonist, SR141716A [78]. On the other hand, CBD, WIN-55,212–2 or 2-AG, all failed to increase the intracellular release of Ca^2+^, with the CB2R antagonist, SR144528, also failing to reduce the degree of induction of JWH-015-mediated Ca^2+^ flux in large DRG neurons. Additional GPR55-HEK293 transgenic studies showed that the inverse agonist of CB1R, AM251, and l-α-lysophosphatidylinositol (LPI; a ligand of GPR55), evoked GPR55-mediated Ca^2+^ oscillation [81]. The endogenous lysophospholipid, LPI, is a well-established mitogenic mediator of oncogenic signalling [82,83]. LPI binding to GPR55 drives the proliferation of cancer cells, often in an autocrine fashion (reviewed in [84]), via the activation of the G protein-RhoA/Rho kinase (Rho/ROCK)-PLC signalling axis, resulting in intracellular Ca^2+^ release from the endoplasmic reticulum (ER). This leads to the activation and subsequent nuclear translocation of the transcription factor NFAT (nuclear factor of activated T-cells), which in turn regulates the transcriptional activity of a number of genes [81]. LPI-mediated Ca^2+^ release was abolished using the Rho/ROCK inhibitor, Y-27632, or by transiently transfecting GPR55-HEK293 cells with a dominant-negative mutant of *RhoA* [81].

These ground-breaking studies have laid the foundations for future research characterising the role GPR55 plays in cancer cell proliferation and anchorage-independent growth [85], angiogenesis [86], migration [87] and metastasis [88,89], with evidence that LPI-induced GPR55 activation plays a role in prostate, ovarian, GBM, breast, skin and pancreatic cancers (reviewed in [84]). Indeed, LPI (10 μM) induced activation of GPR55 in prostate and ovarian cancer cell lines drives the rapid intracellular release of Ca^2+^ to increase phosphorylation of the extracellular signal-regulated kinases, ERK and Akt [85]. Topically, LPI-induced ERK signalling was reduced by pre-treating human ovarian and prostate cancer cell lines with CBD (3.0 μM/0.94 mg/L), or SR141716A (2.0 μM), the Rho/ROCK inhibitor Y-27632 (20 μM), or via the molecular inhibition of *GPR55* [84]. Interestingly, unlike CBD and SR141716A treatments, the inhibition of Rho/ROCK did not reduce LPI induced Ca^2+^ oscillation [84]. This finding recognises an important role for CBD in cancer cell types characterised by high GPR55 activity.

Elevated GPR55 gene expression in breast cancer is associated with reduced disease-free survival, overall survival, and metastasis-free survival, with the highest levels of expression observed in aggressive basal/triple-negative breast tumours [89]. Pro-metastatic properties in this context were exacerbated through an LPI-induced GPR55-G_q/11_ coupling, and activation of RhoA, leading to actin cytoskeleton remodelling, changes in cell dynamics, and increased metastasis. Further, ERK activation in turn stimulates the expression of the transcription factor, ETV4/PEA3 [89] (Figure 1); a gene expression pattern associated with a higher risk of distant metastasis in basal/triple-negative breast cancers [90]. Importantly, previous studies have shown that CBD (5 mg/kg) inhibits the growth of MDA-MB-231 breast cancer cells in vivo using xenograft mouse models [13], once again highlighting a potential role for CBD in the treatment of GPR55 overexpression and aggressive cancer types.

Recently, using the KPC mouse model of pancreatic ductal adenocarcinoma (PDAC), Ferro et al. demonstrated that GPR55 signalling could enhance pancreatic cancer cell growth and proliferation in vivo, via the activation of the mitogen-activated protein kinase (MAPK) signalling pathway (Figure 1) [91]. The knockdown of *GPR55*, or its pharmacological inhibition using CBD, reduced ERK and ribosomal protein S6 phosphorylation, to decrease anchorage-dependent and -independent growth. In addition, inhibition of GPR55 using CBD decreased cyclin D1 and D2 expression and activated the tumour suppressor retinoblastoma (RB) to block cell cycle progression (Figure 1) [91]. The standard of care chemotherapy for pancreatic cancer centres on the use of the nucleoside analogue gemcitabine (GEM), which has previously been shown to increase ERK activation, a suggested mechanism of acquired resistance [92]. GEM can act via inhibition of the enzyme ribonucleotide reductase 1 (RRM1), and resistance to GEM can also be associated with increased RRM1 and RRM2 expression (Figure 1). The expression of both reductases however, was shown to reduce following CBD treatment in vitro [91]. Importantly, treatment using CBD (100 mg/kg), to inhibit GPR55, in combination with GEM (100 mg/kg), improved the survival rate of a PDAC mouse model by a factor of three (mean 52.7 vs. 18.6 days, median 56 vs. 20 days), compared to the vehicle control, and also longer than mice given GEM alone (mean 52.7 vs. 27.8 days, median 56 vs. 23.5). Furthermore, this combined treatment approach, when applied in vitro and in vivo, was also demonstrated to counteract the development of resistance to GEM [91], offering an exciting clinical prospect for the application of CBD in this setting.

#### 3.2.3. Orphan G-Protein-Coupled Receptors GPR3, GPR6 and GPR12

Recently, it was shown that the constitutively active orphan GPCRs; GPR3, GPR6 and GPR12 (GPCRs phylogenetically related to the CBRs, CB1R and CB2R), are novel and specific targets of CBD [93]. These receptors are predominantly expressed in the brain and reproductive tissues, with the binding of CBD to GPR3, GPR6 and GPR12 thought to act as an inverse agonist of the unknown natural ligand of these orphan receptors [93,94]. GPR3 is overexpressed in breast cancer stem cells (CSCs). The inhibition of GPR3 activity in CSCs has been demonstrated to result in the enhanced expression of the transcription factor encoding loci, *NANOG*, and *OCT3/4* [95]. Both NANOG and OCT3/4 play central roles in promoting the self-renewal and long-term proliferative potential of stem-like cancer cells. It is therefore possible that CBD out-competes the endogenous ligand of these orphan GPCRs, thereby blocking β-arrestin2 recruitment to GPR3 and GPR6 [93,94]; an obstruction that would significantly decrease cAMP accumulation and reduce PKA signalling to inhibit the motility, growth and metabolism of the cell [96] (Figure 1). Conversely, CBD may block natural orphaned GPCR signalling, potentially leading to the enrichment of chemotherapy-resistant breast CSCs. Therefore, future research is required to adequately functionally characterise whether the interactions between CBD and these novel receptors, does in actual fact, portend an effective anti-cancer prospect.

### 3.3. Crosstalk between GPCRs and Non-GPCR Signalling Pathways

**Figure 2:**
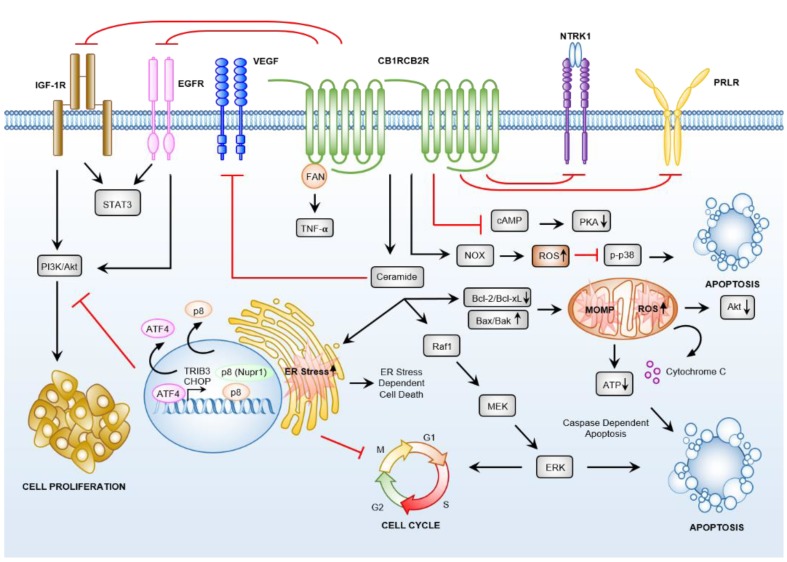
Cannabinoid induced anti-cancer crosstalk between G-protein-coupled receptor (GPCR) and non-GPCR signalling pathways is significant and complex. Cannabinoids interact with CB1R and CB2R and with non-cannabinoid receptors (CBRs) GPCRs (discussed in Section 3.3) to induce cancer cell death via promotion of cellular stress response pathways, increased ceramide synthesis, and reduced oncogenic growth and proliferation signalling.

There is clear evidence that cannabinoids, particularly ∆^9^-THC, modulate the activity of GPCRs, including the well characterised CB1R and CB2R. However, conjecture remains about which downstream pathway(s) elicit the anti-cancer activity of CBD. While most studies have demonstrated that CBD has low affinity for CB1R and CB2R, research by McKallip et al. and Massi et al. revealed that in lymphoma and acute lymphoblastic leukaemia (ALL) cell lines and human glioma cell lines, respectively, CBD (2.5 µM/0.8 mg/L) induced apoptosis through a CB2R-specific mechanism, and not via binding to either CB1R or TRPV1 [51,97] (Figure 2). Furthermore, the treatment of primary T-ALL lymphoblasts with the CB2R selective agonist, JWH-133, and of Jurkat cells with the TRPV1 agonist, RTX, also induced apoptosis [98]. Whether the CBD effects in this context are a result of the level of CBR activity in these cells is yet to be unequivocally determined. However, additional in vitro research has shown that CBD inhibits human breast cancer cell growth and induces apoptosis via the activation of both CB2R and TRPV1 [13]. Taken together, these findings put forth the hypothesis that crosstalk between the GPCRs and non-GPCR signalling pathways may direct the selective anti-cancer activity of cannabinoids.

Further evidence of crosstalk between oncogenic signalling pathways and either CB1R or CB2R is highlighted by the observation that ∆^9^-THC, WIN-55,212-2 or JWH-133, can downregulate the vascular endothelial growth factor receptors (VEGFRs), to reduce the angiogenesis and metastasis of skin cancer cells in vivo [99], and of glioma cells in vitro and in vivo [100] (Figure 2). The eCBs, AEA and 2-AG, and the sCB, HU-210, inhibit the proliferation of human breast and prostate cancer cells via crosstalk between the CB1R, the high-affinity nerve growth factor receptor (NTRK1) and the prolactin receptor (PRLR) signalling pathways [53] (Figure 2). In oestrogen receptor-α negative (ERα-) and positive (ERα+) breast cancers, the activation of CB2R by JWH-015 reduced breast cancer cell survival via blocking of the EGFR and insulin-like growth factor-1 receptor (IGF1R) pathways. Blockage of the EGFR and IGF1R pathways in turn leads to the reduced activation of the downstream signal transducer and activator of transcription 3 (STAT3), RAC-α,β,γ, Akt and ERK signalling pathways [101] (Figure 2). Interestingly, positive correlation between elevated CB2R expression and relapse-free survival has been established [53]. This positive correlation is particularly evident in ERα+ and ERα- breast cancer patient samples, cancer cell types known to have elevated EGFR and IGF1R expression [53]. Such observations may support the potential use of CB2R agonists as an adjuvant therapy option for ERα+ and ERα- breast cancer patients [101].

CBD has been shown to be effective alone, and in combination with the approved proteasome inhibitor, bortezomib, for the treatment of multiple myeloma (MM) [60]. In this context, TRPV2 was shown to regulate the cytotoxic effects of CBD, in a TPRV2 expression dependent manner [60]. TRPV2 activation by CBD decreased the levels of phosphorylation of ERK and Akt, and synergised the effects of bortezomib treatment, to decrease cyclin D1 (*CCND1*) expression. More recent studies highlighted the crosstalk between CBR-dependent and independent anti-cancer effects induced by cannabinoids in MM. For example, Nabissi et al. further revealed that CBD was able to arrest the growth and survival of MM cell lines by halting the cell cycle at the G1 phase, but also showed that Δ^9^-THC was an additional effective cell cycle inhibitor, a finding that further highlights a role for Δ^9^-THC in TPRV2 activation [61]. These studies showed that a 1:1 combination of CBD and ∆^9^-THC was statistically more effective at increasing the G1 cell population, compared with either CBD or Δ^9^-THC treatment alone [61], effects not antagonised via the use of the CB2R antagonist, AM630. Interestingly, rather than activating apoptotic cascades, autophagic cell death was induced following combination treatment with Δ^9^-THC and CBD. These studies went on to show reduced migration and down-regulation of the chemokine receptor, CXCR4, and of the CD147 plasma membrane glycoprotein, using CBD or Δ^9^-THC alone (CBD 12.5 μM/3.9 mg/L, Δ^9^-THC 12.5 μM/3.9 mg/L), Δ^9^-THC:CBD in combination, the immuno-proteasome inhibitor carfilzomib (100 nM) alone, and the combined treatment with all three compounds. The results of these preclinical studies have recently prompted investigators to commence a randomised, double-blind, placebo-controlled clinical trial to investigate whether combination of CBD with bortezomib will have greater anti-tumour and anti-proliferative activity compared to standard of care (bortezomib alone) in MM, GBM and gastrointestinal (GI) malignancies (Table 2).

The crosstalk between non-THC cannabinoids and the modulation of the GPCR-dependent, and GPCR-independent signalling pathways, is becoming more evident. However, there remains an urgent need for the application of a more focused research approach that are based on the use of purified non-THC cannabinoids and the ‘simulated’ assessment of downstream effects using whole proteome data [102], with the elegant supplementation of phosphoproteome data [103]. The heterogeneity of cancer is such that, until the field acquires a more detailed understanding of the downstream effects of both ∆^9^-THC and non-THC cannabinoids, unlocking the definitive and selective anti-cancer mechanisms of action of these compounds will remain ambiguous, and continue to limit their clinical utility.

### 3.4. Cannabinoid Receptor-Dependent Cancer Effects

**Figure 3:**
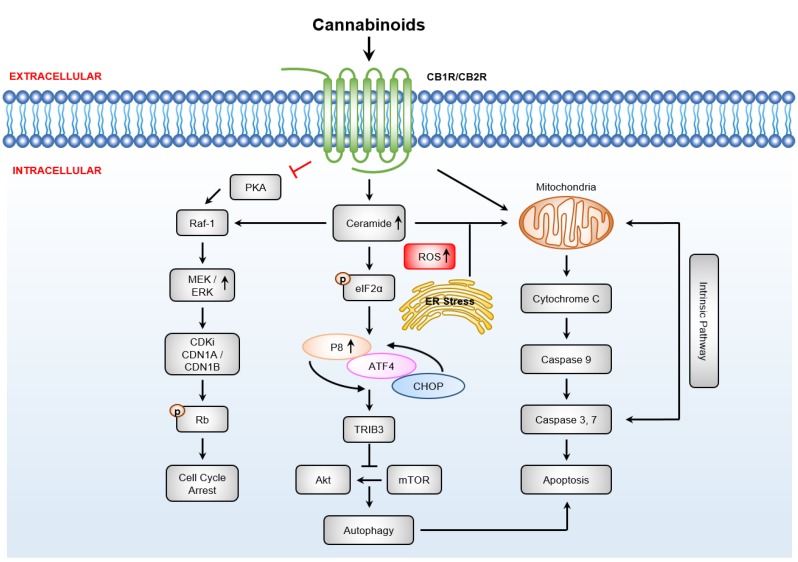
Anti-cancer effects and cell death pathways regulated by cannabinoids and the classical cannabinoid receptors. The activation of cannabinoid receptors (CBRs) drives sustained extracellular signal-regulated kinases (ERK) activity driving reactive oxygen species (ROS) production and leads to activation of cyclin kinase inhibitors resulting in G1 cell cycle arrest [45,115]. Agonism of cannabinoids receptors increases ceramide synthesis to induce endoplasmic reticulum (ER) stress driving phosphorylation and activity of the transcription factor eukaryotic translation initiation factor 2A (eIF2α), leading to upregulation of p8 and its downstream targets cyclic AMP-dependent transcription factor ATF-4 (ATF4), C/EBP homologous protein (CHOP), and tribbles homolog 3 (TRIB3). Activation of p8 inhibits the Akt/mTORC1 axis, driving autophagy-mediated cell death pathway upstream of apoptosis [116].

There is growing evidence that cannabinoids inhibit tumour growth and metastasis and induce tumour-specific apoptosis in cancer cells following the activation of CB1R or CB2R (Figure 3) [39,47,104]. Although counterintuitively, it is generally recognised that high grade gliomas, such as GBMs, express high levels of CB2R, with the degree of CB2R expression correlating with tumour grade (reviewed in [105]). However, the data remains unclear for CB1R. In GBM tissue, compared to low-grade gliomas, or non-tumour control tissue, De Jesús et al. revealed reduced expression of CB1R [106], Schley et al. determined that CBR1 expression remained unchanged [107], and Wu et al. [108] and Ciaglia et al. [109] found CBR1 expression to be elevated (reviewed in [110]). In the most aggressive and lethal of all children’s cancers, diffuse intrinsic pontine glioma (DIPG), also known as diffuse midline glioma (DMG), the picture is even more unclear [111]. The abundance and/or activity of these two receptors is more clearly reported in astrocytoma, meningioma, non-small-cell lung carcinoma (NSCLC) and lymphoma, as well as skin, prostate, breast and colon cancers (reviewed in [14]). Taken together, these findings potentially highlight the use of ∆^9^-THC and CBD to elicit cancer selective cell death via CBR-dependent and -independent mechanisms, respectively. However, there is a distinct difference between the stimulatory and inhibitory activities of these receptors, a difference that predominantly depends on the agonist, and on the cancer cell line used [112]. The binding of ∆^9^-THC to CB1R or CB2R reduces cancer cell survival by inducing a stress response driving ceramide production, which in turn modulates growth and proliferation signal transduction cascades, to activate both extrinsic and intrinsic caspases (Figure 3) [47,112]. The CB1R and CB2R agonist, ∆^9^-THC, inhibits cell growth by sustained ceramide accumulation, and via the activation of ERK, c-Jun N-terminal kinase (JNK) and p38 MAPK [69,113]. Treatment with CBR agonists, WIN-55,212-2, JWH-015 and JWH-133, also inhibits the growth and metastasis of NSCLC in vitro and in vivo by inhibiting Akt phosphorylation and reducing matrix metalloproteinase-9 (MMP9) expression and activity. This in turn inhibits the reorganisation of the extracellular matrix, as well as to also inhibit the migratory potential of malignant cells [114].

As alluded to in Section 1, ‘not every cloud has a silver lining’, with ∆^9^-THC exposure demonstrated to promote mouse mammary tumour growth and metastasis, due to its role in reducing the specific anti-tumour immune responses of immune cells known to express high levels of CB2R [8]. Importantly, the agonism of CBRs using nanomolar concentrations of ∆^9^-THC increased the proliferation of cancer cells through activation of MAPK via transactivation of EGFR [117]. These pro-survival (cancer propagating) effects are also seen for AEA and the sCBs, HU-210 and WIN-55,212-2 [117]. The concentration of ∆^9^-THC in this context is likely to reflect serum levels detected after ∆^9^-THC administration and hence are therapeutically relevant. At the current time, the prosurvival effects seen for ∆^9^-THC have not been reported for CBD. Whether this is a result of lack of CBD affinity for CBRs, particularly in immune cells, or whether pro-survival signalling requires CBR-induced transactivation of receptor tyrosine kinases, activation that is not possible following the administration of CBD due to CBD lacking affinity for CBRs, remains to be tested.

#### 3.4.1. Cannabinoids Induce Apoptosis via Sustained Ceramide Accumulation

Numerous reports have shown that the modulation of CBR activity by cannabinoids inhibits the in vitro and in vivo growth and survival of cancer cells via the production of ceramide, a pro-apoptotic sphingolipid [49,69,118,119,120]. CBR activation by either ∆^9^-THC or WIN-55,212-2 drives the intracellular accumulation of ceramide to activate Raf1/ERK signalling, which in turn leads to the production of damaging cellular reactive oxygen species (ROS) (Figure 2 and Figure 3). This drives genotoxic stress and apoptosis in glioma cells, both in vitro and in vivo [69]. Ceramide accumulation leads to ER stress, which in turn enhances the expression of the stress-regulated protein, p8, with enhanced p8 abundance leading to the inhibition of the Akt/mammalian target of rapamycin (mTOR) cell signalling axis and the activation of caspase cascades to drive apoptosis and autophagy [2,121]. Likewise, the increased expression of cellular tumour antigen, p53 (*TP53*), and of the apoptosis regulator, BAX (*BAX*), plus the reduced expression of anti-apoptotic protein Bcl-2, (*BAD*), have all been demonstrated in cervical cancer cells treated with cannabis extracts or purified CBD (Figure 2) [46]. Additional reports show that in human colon cancer cell lines, activation of the individual and specific activities of certain CBRs induces apoptosis through a tumour necrosis factor-α (TNF-α) mediated de novo synthesis of ceramide [49].

#### 3.4.2. Downstream Targets of Ceramide

The ceramide-dependent upregulation of stress-regulated protein, p8, is a hallmark of treatment of glioma, astrocytoma, and pancreatic cancer cells with cannabinoids [119]. ER stress drives phosphorylation and activity of the transcription factor, eIF2α, leading to upregulation of p8 and of the downstream proteins, including cAMP-dependent transcription factor, ATF-4, C/EBP homologous protein, CHOP, and pseudokinase tribbles-homologue 3, TRIB3, which sensitises tumour cells to ∆^9^-THC treatment and leads to cell death via autophagy and apoptosis (Figure 2 and Figure 3) [116,121]. It is important to note here that Akt dysfunction is one of the most common molecular events in cancer. Increased TRIB3 activity inhibits the Akt/mTOR signalling axis to trigger autophagy (upstream of intrinsic mitochondrial apoptosis), and further, decreased Akt activity together with increased BAD activity, directs the activation of cyclin-dependent kinase inhibitor 1A (*CDKN1A*) and *CDKN1B* (reviewed in [2]).

CBD and ∆^9^-THC induced apoptosis in vitro and in vivo using human breast [122] and leukaemic cells [51,120] has been shown to be regulated by ceramide biosynthesis. Ceramide reduces mitochondrial membrane potential by the translocation of BH3-interacting domain death agonist protein, BID, to the mitochondria to release the small hemeprotein, cytochrome C (*CYC1*) to the cytosol, ultimately activating intrinsic apoptotic pathways via the caspase cascade (*CASP8*, *CASP9*, and *CASP3*) [120,123] (Figure 2). The overlap between apoptosis and autophagy is potentially regulated by Beclin-1 (*BECN1*); CBD enhanced the interaction between BECN1 and phosphatidylinositol 3-kinase catalytic subunit type 3 (*PIK3C3*), as this protein-protein interaction cross-regulates the instigation of autophagosomes, and the activation of apoptosis, through direct interactions with anti-apoptosis family members Bcl-2 and/or Bcl-xL (reviewed in [124]). In short, CBR-induced cancer cell death relies on ceramide-induced ER stress and the upregulation of p8 driving caspase cascade activation (Figure 3).

#### 3.4.3. Cannabinoid Induced Anti-Cancer Effects

A trademark effect of the treatment of cancer cells with both the pCBs, CBD and ∆^9^-THC, and the eCBs, AEA and 2-AG, is the production of ROS, resulting in increased cellular oxidative stress [125,126,127]. Indeed, CBD drives ROS production to deplete the intracellular stores of glutathione (GSH), and triggers the activity of GSH-associated enzymes such as glutathione peroxidases and glutathione reductases, to activate the pro-apoptotic proteins, caspase-8 (*CASP8*) and CASP9 (*CASP9*) (Figure 3). Elevated CASP8 and CASP9 abundance within a cancer cell promotes the cleavage of CASP3 (*CASP3*), and this in turn, leads to cell death [97,122,127]. However, supplementation with the antioxidant, α-tocopherol, rescues the cytotoxic effects of AEA and 2-AG [125]. Indeed, treatment of human ALL cells with CBD promotes the expression and activity of the phagocyte nicotinamide adenine dinucleotide phosphate (NADPH) oxidase (NOX) subunit, NOX4 (*NOX4*), and its regulatory protein, p22^phox^ (*CYBA*) [51]. Activated NOX4 is responsible for directing the ‘respiratory burst’ (the rapid release of ROS) required for the destruction of invading microbes (reviewed in [128]). CBD-induced ROS production, and hence increased oxidative stress, decreases the phosphorylation status of p38 MAPK to direct apoptosis (Figure 2). In line with these observations, treatment of ALL cells with the CB2R-selective antagonist, SR144528, NADPH oxidase inhibitors, or ROS scavengers, significantly attenuated apoptosis induced by CBD [51]. In direct contrast, ∆^9^-THC used in animal models to study the human immunodeficiency virus (HIV), decreased NOX4 expression, which in turn reduced the levels of cellular oxidative stress in intestinal cells, via upregulation of the abundance of the *NOX4* targeting microRNA, miR-99b, thereby decreasing the rate of translation of the NOX4 protein [129]. In addition, Olivas et al. showed that in T-ALL cells sensitive to CBD, cell death was not influenced by CBRs [130]. Autophagy was activated by sub-lethal CBD concentrations by Ca^2+^ overload in the mitochondria, releasing cytochrome-C, and potentially highlighting a role for GPR55 in this context. It is well documented that the anti-inflammatory effects of ∆^9^-THC in the gastrointestinal tract are predominantly mediated by modulating CB2R activity, and therefore, the pro-oxidative effects of CBD may not strictly be the result of the antagonism of CB2R, but of crosstalk, or independent activation of non-CBRs GPCRs or ion channels.

Apoptosis induced by CBD in lung cancer cells has also been shown to be independent of CB1R or CB2R activity [52]. CBD treatment-induced expression at both the mRNA and protein level of the nuclear receptor, and transcription factor, peroxisome proliferator-activated receptor gamma (PPAR-γ (*PPARG*)) and of the cyclooxygenase-2 (COX-2 (prostaglandin G/H synthase 2–*PTGS2*)) transcript/protein [130]. Apoptosis was abrogated by the use of COX-2 inhibitors and PPAR-γ antagonists in lung cancer cell lines, primary lung cancer patient samples, and in vivo, using athymic nude mice xenografted with the lung cancer cell line A549 (Figure 2). CBD has been shown to directly bind PPAR-γ to increase its transcriptional activity [131]. These findings suggest that COX-2 and PPAR-γ are critical for the anti-cancer effects of CBD in lung cancer and provide strong evidence that the molecular targets of CBD may at least be, in part, dependent on the cancer cell type being treated.

## 4. Clinical Evidence: Cannabis for Use by Patients with Cancer

Pre-clinical studies investigating the potential anti-cancer properties of cannabis (and of cannabinoids) have rekindled clinical interest in their use for the treatment of various cancer types (Table 2) [110,132]. Whilst significant in vitro and in vivo research is currently underway, there are only a limited number of clinical trials investigating the potential of low-THC cannabis or non-THC cannabinoids for the treatment of cancer. That being said, the long-term risk-benefit ratio for the use of CBD is yet to be determined, especially in the adolescent population. Research on cannabinoid:drug interactions are also urgently needed and will provide the necessary pharmacokinetic and toxicological parameters for future treatment strategies.

In an uncontrolled retrospective case-series of 119 breast cancer and/or glioma patients who received synthetic, pharmaceutical-grade CBD alone, or in combination with other treatments, synthetic CBD was well tolerated and not limited by a maximal tolerated dose. Measures of anti-cancer efficacy included a reduction in circulating tumour cells or a radiological response, and while most claims of benefit could be related to interpatient variation or cancer heterogeneity, occasional extraordinary responders were noted, including two patients with ependymoma [132]. The average dose of CBD was 10 mg twice daily, administered via a three day on/three day off rotation. Such an administrative approach was achieved in the complete absence of any notable side effects. An extended median overall survival rate of 36% was observed for patients on this regimen, with 24% of these patients administered CBD only. A separate group of nine GBM patients received a daily dose of 400 mg CBD (approximately 6.5 mg/kg), alongside standard of care including maximal resection followed by radiochemotherapy [133]. Of the nine GBM patients in this treatment group, eight patients were alive with a mean survival of 22.3 months, almost two-fold the median overall survival for GBM patients.

Additionally, an unpublished and underpowered phase II trial study in recurrent GBM patients treated with a 1:1 ratio of ∆^9^-THC:CBD (27 mg/mL and 25 mg/mL) in combination with temozolomide, reported an increased 1-year overall survival rate of 83%, compared to 44% for patients treated with temozolomide alone (*p* = 0.042) (reviewed [110]). Such a promising result was accompanied by treatment emergent adverse events, including vomiting (75%), dizziness (67%) nausea (58%), headache (33%), and constipation (33%), compared to limited, or no, adverse events in emerging clinical investigations using CBD alone [132].

## 5. Role of Non-THC Cannabinoids in Cancer-Associated Inflammation

The relationship between inflammation and the evolution of certain cancer types is thoroughly established. Up to 20% of cancer associated deaths worldwide are related to cancer-induced inflammation [67]. There is emerging evidence to suggest that cannabinoids induce apoptosis of inflammatory, or of other immune cell types, via CBR activation. Ligation of cannabinoids with CB2R has been demonstrated to function as a potent regulator of immune responses due to its abundance in all immune cell subtypes, including B cells, T cells and NK (natural killer) cells [67]. Agonist binding to CB2R drives immunosuppression through the inhibition of adenylate cyclase (AC), which in turn, downregulates the cAMP signal transduction pathways and the activity of cAMP-dependent kinases (PKA) [134,135]. Likewise, in human monocytes and macrophages, CB2R activation directs the decreased production of the pro-inflammatory cytokines, interleukin-6 (IL-6), IL-8, IL-12 and TNFα [134]. Elbaz et al. reported that breast cancer cells treated with CBD have reduced levels of inflammatory cytokines, such as C-C motif chemokine 3 (*CCL3*), granulocyte-macrophage colony-stimulating factor GM-CSF (*CSF2*), and macrophage inflammatory protein 2 (MIP-2), as well as decreased recruitment of macrophages to the tumour microenvironment [136]. Similarly, CBD has exhibited protective effects in intestinal inflammation and has been demonstrated to inhibit the release of cytokines and to promote wound healing [137].

It is important to consider the immunomodulatory effects of cannabinoids when considering their combined use with anti-cancer immunotherapies, including the checkpoint inhibitors (anti-CTLA-4 and anti-PD-1/PD-L1), or with cellular therapies such as chimeric antigen receptor T-cells (CAR-T), particularly for regimens including ∆^9^-THC. A recent retrospective, observational study of patients with either advanced melanoma (NSCLC), or renal cell carcinoma, showed that study participants who received nivolumab (an anti-PD-1 therapy), in combination with an unspecified cannabis product, had lower response rates (RR) to nivolumab, compared to those receiving nivolumab alone (i.e., 15.9% compared to 37.5%; odds ratio [OR] 3.13, *p* = 0.016) [9]. Interestingly, patients who included cannabis as part of their immunotherapy treatment regimen showed no significant differences in either progression-free survival (*p =* 0.27) or overall survival (*p* = 0.45) [9], even though this patient cohort did not respond to nivolumab. This finding presents a curious question, that being; why did cannabis patients that failed to respond to immunotherapy, survive just as long as those patients who responded to immunotherapy? Deciphering the clinical benefit from the use of cannabinoids through the inclusion of a ‘cannabis/cannabinoid treatment’ alone, or ‘cannabis in combination with standard-of-care chemotherapies’ may greatly aid in the clinical utilisation of cannabis when targeting the immune system as a frontline treatment strategy.

## 6. Conclusions

There is a clear imperative to improve the treatment of cancer, particularly for cancers lacking effective treatment options. New immunotherapeutic approaches are dramatically increasing the survival of patients with advanced disease states, such as those patients with metastatic melanoma; where previously survival was measured in months. However, sophisticated immunological or cell-based treatment approaches are yet to be developed (or are not always viable) for high-risk or advanced cancers, including relapsed acute myeloid leukaemia (AML) [138], or high-risk T-ALL [103]. Similarly, paediatric patients with highly aggressive brainstem cancers, such as DIPG or DMG, progress soon after upfront radiotherapy. This standard of care regimen is accompanied by peritumoural inflammation and hydrocephalus [111], and represents a patient cohort with greatly unmet therapeutic needs: the opportunity to employ complementary therapeutics (that specifically lack psychotropic side effects) to enhance or complement standard of care management of high risk, aggressive cancers warrants intensive investigation.

Several pre-clinical and clinical investigations have focused on the anti-cancer potential of cannabis and its constituents, namely CBD and ∆^9^-THC, and are beginning to reveal novel therapeutic potentials. Nevertheless, in addition to social and legal complexities, the serious side effects associated with the use of ∆^9^-THC (particularly in children and elderly patients) are a major roadblock in the use of cannabis in the medical setting. Consequently, research and consumer interest in low-THC cannabis can deliver an alternative to traditional cannabis, including an alternate treatment route (oral dosing), and has gained considerable momentum recently. Unfortunately, research focused on the anti-cancer properties of low-THC cannabis, and of its major pharmacologically active components, such as CBD, are slow to reach clinical studies. Most of the available literature remains limited to reports of the pre-clinical efficacy of low-THC cannabis. As a result, a comprehensive and clear picture of the mechanisms driving the anti-cancer properties of low-THC cannabis, and of non-THC cannabinoids, remains a work in progress.

Consistent lines of evidence show that cannabinoids play a role in modulating neural and immune functions, both centrally and peripherally, via the classical CB1R and CB2R mediated receptor routes, particularly for ∆^9^-THC, while CBD is more commonly reported to function as a ligand for other GPCRs (including GPR55, GPR3, GPR6, GPR12, TRPVs and PPAR-γ). While the exact mechanistic basis remains unclear, there appears to be significant crosstalk between the molecular targets of cannabinoid responsive pathways, targets which could ultimately determine downstream proliferation or anti-tumour responses. If this was not complicated enough; the use of cannabis plant extracts, which are generally highly complex mixtures of cannabinoids, terpenoids and other bioactive natural products, makes identification of the anti-cancer benefits even more challenging. That being said, studies generally show that pure CBD is superior to CBD-rich extracts and other plant cannabinoids (CBG, CBC) for inhibition of tumour cell growth and survival, and without any cytotoxic effect on healthy cells, at the doses reported (IC50: 6–10 µM/1.9–3.1 mg/L) [139]. While the application of low-THC cannabis extracts may prove beneficial as an alternate cancer treatment in the future, owing to the potential cooperative nature of its diverse chemical components (entourage effect), conversely, it could also undermine efficacy and foster the development of drug resistance to established chemotherapeutic protocols. More data is still urgently needed to establish the ‘pros’ and ‘cons’ of using whole cannabis extracts, or isolated cannabinoids, before its application in clinical trial settings.

There is a strong and compelling case for further mechanistic studies into the potential anti-tumour and related health benefits of low-THC cannabis. Proteogenomic studies [140], and studies focused on cellular responses to cannabinoids [141], will ultimately provide mechanistic data to determine whether CBD and other non-THC cannabinoids can be included as safe, alternative treatment strategies to those currently investigating ∆^9^-THC-based strategies in the clinic. Given that a number of the chemical components of low-THC cannabis interact with several critical molecular targets, and that these targets repeatedly demonstrate an altered expression and/or accumulation profile in cancerous cells, further research into the anti-cancer properties of medicinal cannabis, could lead to the discovery of effective treatments and deliver further invaluable insight into cancer cell biology for researchers, clinicians and patients alike.

## Figures and Tables

**Table 1:** Anti-cancer effects of cannabinoids in various cancer cell lines.

Cancer Type	Model/Cell Line	Compound	Effective Dose	Effects	References
Breast	MCF-7	Cannabidiol (CBD)	8.2 µM/2.6 mg/L	Inhibition of cancer cell growth and proliferation	[13]
		CBD-rich extract (~70% CBD)	6.0 µM/2.7 mg/L		
		∆^9^-tetrahydrocannabinol (∆^9^-THC)	14.2 µM/4.5 mg/L		
		AEA	1.4 µM/0.5 mg/L		[53]
	MDA-MB-231	CBD	2.2 µM/0.7 mg/L	Inhibition of cancer cell growth, induction of apoptosis	[54]
		WIN-55,212-2, JWH-133	10 µM/4.3 mg/L, 10 µM/3.1 mg/L	Inhibition of proliferation	[55]
	Xenograft-MBA-MD-231 cells	CBD	5 mg/kg (i.p.)	Reduced tumour size and volume	[13]
		CBD-rich extract (~70% CBD)	6.5 mg/kg (i.p.)		
		WIN-55,212-2, JWH-133	5 mg/kg (i.p.)	Reduced tumour growth, angiogenesis and metastasis	[55]
Cervical	SiHa	CBD	3.2 µg/mL/3.2 mg/L	Inhibition of cancer cell growth, induction of apoptosis	[46]
	HeLa		3.2 µg/mL/3.2 mg/L		
	ME-180		1.5 µg/mL/1.5 mg/L		
Colon	HCT8	CB-13	>50 nmol/L/0.02 mg/L	Inhibition of cancer cell growth	[49]
	SW480				
	HCA7				
	HCT15				
	HCT-116	CBG	≥3.0 µM/0.9 mg/L	Reduced viability of cancer cells	[56]
	Xenograft-HCT-116	CBG	3 and 10 mg/kg (i.p.)	Inhibition of tumour growth	
Glioma	U251	CBD	0.6 µM/0.2 mg/L	Inhibition of cancer cell growth	[47]
		∆^9^-THC	3.3 µM/1 mg/L		
	U87	CBD	0.6 µM/0.2 mg/L		
		∆^9^-THC	3.3 µM/1 mg/L		
	GSC3832	CBD	3.5 µM/1.1 mg/L	Inhibition of viability	[57]
	GSC387	CBD	2.6 µM/0.8 mg/L		
	U87MG	CBD	>25 µM/>7.9 mg/L	Reduced viability and induce cancer cell death	[58]
	Xenograft-U87	CBD	6.7 mg with 75 mg micro-particles	Inhibition of tumour growth	[59]
Multiple Myeloma	U266	CBD	32.2 µM/10.1 mg/L	Reduced cancer cell viability, increased cytotoxicity, inhibition of cancer cell migration	[60,61]
		∆^9^-THC	39.5 μM/12.4 mg/L		
		CBD + ∆^9^-THC + carfilzomib	(0–50 μM/0–15.7 mg/L) CBD + (12.5–50 μM/3.9–15.7 mg/L) ∆^9^-THC + (12.5–100 nM/0.009–0.072 mg/L) carfilzomib		
	U266_TRPV2_	CBD	19.8 μM/6.2 mg/L		
	RPMI	CBD	22.4 μM/7.0 mg/L		
		∆^9^-THC	30.8 μM/9.7 mg/L		
		CBD + ∆^9^-THC + carfilzomib	(0–50 μM/0–15.7 mg/L) CBD + (12.5–50 μM/3.9–15.7 mg/L) ∆^9^-THC + (0.9–7.5 nM/0.0006–0.005 mg/L) carfilzomib		
	RPMI_TRPV2_	CBD	13.5 μM/4.2 mg/L		

**Table 2:** Clinical trials using cannabinoids for the treatment of cancer *.

Trial No.	Cancer Type/s	Study Type/Phase	Treatments	Dose of Cannabinoids or Cannabis Products	Delivery	Outcome ^ƛ^
NCT01812603; NCT01812616	Glioblastoma multiforme (GBM)	Interventional (Clinical Trial)/Phase 1 & Phase 2	Combination of Temozolomide (TMZ) and Sativex (1:1 ∆^9^-THC:CBD)	Dose-intense TMZ with a maximum of 32.4 mg THC and 30 mg CBD per day	Oral spray	Increased 39% of 1-year survival rate
NCT02255292	Solid tumour	Interventional (Clinical Trial)/Phase 2	CBD	Unknown	Unknown	Not yet recruiting
NCT01489826	Solid tumour	Interventional (Clinical Trial)/Phase 1	Dexanabinol (HU-211; a synthetic cannabinoid)	2–36 mg/kg once weekly–3 doses in 21-day cycle	Intravenous infusion	Progression-free survival increased
NCT01654497	Brain cancer	Interventional (Clinical Trial)/Phase 1	Dexanabinol (HU-211)	2–44 mg/kg once weekly–4 doses in 28-day cycle	Intravenous infusion	No relevant results available
NCT03431363	Head and neck cancer	Observational	Medically certified cannabis with adjuvant chemoradiation	Dosing options to be stratified into 3 groups viz. standard, frail/elderly (age > 65 or ECOG 2), and cannabis-experienced	Smoke	Recruiting
NCT02423239	Hepatocellular carcinoma; pancreatic cancer	Interventional (Clinical Trial)/Phase 1	Dexanabinol (HU-211) monotherapy and in combination with chemotherapy	MTD ** once a week	Intravenous infusion	Ongoing
NCT03245658	Pancreatic cancer	Interventional (Clinical Trial)/Phase 2	1:2 ∆^9^-THC:CBD	Individually titrated doses on daily basis; for 4 weeks	Oral drops	Not yet recruiting
NCT03529448	GBM	Interventional (Clinical Trial)/ Phase 1 & Phase 2	TN-TC11G (1:1 ∆^9^-THC:CBD) combination with temozolomide and radiotherapy	Total daily dose of 10–160 mg, after meal	Unknown	Not yet recruiting
NCT03617692	Non-small-cell lung carcinoma (NSCLC) metastasis	Observational	Cannabis products	Products, dose and administration frequency decided by study participants	Oral administration	Recruiting
NCT03052738	Paediatric CNS tumour	Observational	Medical marijuana-derived products	Method of delivery, strain used, dosing and frequency decided by study participants		Recruiting
NCT03687034	Glioblastoma	Interventional (Clinical Trial)/Phase 1	CBD with standard of care	Escalating doses of CBD	Oral sublingual formulation	Not yet recruiting
NCT03607643	GI malignancies (pancreas, liver rectum, colon, or gall bladder), multiple myeloma, or GBM	Interventional (Clinical Trial)/Phase 1 & Phase 2	CBD with standard of care chemotherapy	100 mg twice daily before meal	Oral sublingual formulation	Not yet recruiting
ACTRN12617001287325	GBM	Interventional (Clinical Trial)/Phase 2	1:1 ∆^9^-THC:CBD (6 mg/mL:6 mg/mL) or 1:4 CBD:∆^9^-THC (3.8 mg/mL:15 mg/mL) and standard treatment ***	Starts at 0.25 mL at night and each night titrated up or downwards by 0.05 mL based on participant’s response	Oral oily liquids	No relevant results available
ACTRN12619000265178	Any cancer	Interventional (Clinical Trial)/phase 4	∆^9^-THC or 1:1 ∆^9^-THC:CBD. Combined with standard treatment for advanced cancer and symptoms	Starts at 2.5 mg THC three times a day in cannabis naive patients, and 5 mg THC three times a day in previous users. Dosage adjusted based on patient’s response up to a maximum of 30 mg THC per day.	Oral oily liquids	Recruiting
ACTRN12619000037101	Any cancer	Interventional (Clinical Trial)/Phase 2	1:1 ∆^9^-THC:CBD	Total daily dose of 2.5 mg:2.5 mg–30 mg:30 mg	Oral oily liquid	Recruiting
ACTRN12618001220257	Any cancer	Interventional (Clinical Trial)/Phase 2	CBD	Total daily dose of 50 mg–600 mg	Oral oily liquid	Recruiting
ACTRN12618001205224	Any cancer	Interventional (Clinical Trial)/Phase 1	CBD or ∆^9^-THC with palliative care	Total daily doses of 50 mg–600 mg/day for CBD or 2.5 mg–30 mg for THC	Oral oily liquid	Doses of THC and CBD used in the study were generally well tolerated and up to 50% of the participants had an overall improvement in their condition since starting cannabis but results need to be replicated in placebo controlled trial [62]
ACTRN12616001036404	Any cancer	Interventional (Clinical Trial)/Phase 2 & Phase 3	1:1 ∆^9^-THC/CBD with chemotherapy	2.5 mg THC and 2.5 mg CBD, once the day before chemotherapy and three times daily for 5 days for the first 5 days of participants’ chemotherapy cycle and for three consecutive cycles.	Oral Capsule	Recruiting

* Sources: www.clinicaltrials.gov and www.anzctr.org.au (ANZCTR), ** Maximum Tolerated Dose, *** Includes chemotherapy, radiation, immunotherapy, or any other cancer related treatment requested by the participants medical specialists and including best supportive and palliative care, ^ƛ^ As at 3 April 2020.
